# Proteomic analysis confirmed that the occurrence of diabetic sarcopenia is related to autophagy and apoptosis

**DOI:** 10.3389/fendo.2025.1656035

**Published:** 2026-01-05

**Authors:** Tianrui Wang, Liang Zhang, Yu Jiang, Xia Zhao, Kehao Hou, Yingze Zhang, Ning Yu, Kuishuai Xu

**Affiliations:** 1 Knee Preservation Center, the Affiliated Hospital of Qingdao University, Shandong, Qingdao, China; 2 Department of Abdominal ultrasound, Affiliated Hospital of Qingdao University, Shandong Qingdao, China; 3 Department of Sports Medicine, the Affiliated Hospital of Qingdao University, Shandong, Qingdao, China

**Keywords:** apoptosis, autophagy, gastrocnemius muscle, proteomics, type 2 diabetes mellitus

## Abstract

**Objective:**

More and more evidence indicates that type 2 diabetes mellitus (T2DM) can cause muscle lesions and lead to the occurrence of sarcopenia. The changes in the gastrocnemius muscle of T2DM rats were investigated by using tandem mass tag (TMT) proteomics technology, with the aim of discovering biomarkers of muscle lesions induced by T2DM and clarifying their potential biological relationships.

**Methods:**

Twelve healthy male Sprague Dawley rats were randomly divided into the Control group (n=6) and the T2DM group (n=6). The experimental group was established by intraperitoneal injection of streptozotocin at a dose of 40mg/kg combined with a high-fat and high-sugar diet, to create a T2DM rat model. Six months later, the gastrocnemius muscles of the two groups of rats were selected for histological staining and proteomic analysis. Immunohistochemical staining, WB and qRT-PCR were used to verify the expression of proteins related to autophagy and apoptosis.

**Results:**

The results of HE staining showed that the gastrocnemius muscles of T2DM rats were atrophied, with a reduced cross-sectional area, increased cell spacing, and signs of hemorrhage and inflammatory infiltration. The immunohistochemical staining results indicated that the expression levels of proteins related to muscle atrophy (MAFbx and Ube2b) in the gastrocnemius muscles of T2DM rats were significantly increased (P < 0.001). These histological changes were consistent with the pathological and physiological manifestations of sarcopenia. The results of proteomics revealed that 273 differentially expressed proteins (DEPs, 133 upregulated and 140 downregulated) were identified in the gastrocnemius muscles of T2DM rats. KEGG enrichment analysis showed that the DEPs were involved in 94 signaling pathways, including autophagy and apoptosis. After verifying the key proteins and genes in the autophagy and apoptosis pathways, it was found that the expressions of p62, Bax and Caspase-3 in the gastrocnemius muscles of T2DM rats significantly increased, while the ratio of LC3II/I significantly decreased. These results indicate that the autophagy level in the gastrocnemius tissues of T2DM rats significantly decreased, and the cell apoptosis significantly increased.

**Conclusions:**

This study employed proteomics techniques to conduct bioinformatics analysis of DEPs in the gastrocnemius muscles of T2DM rats, as well as to explore potential pathogenic pathways. By verifying the proteins related to the pathways of autophagy and apoptosis in cells, we have, for the first time, confirmed through proteomics data that sarcopenia caused by T2DM may be associated with decreased autophagy and increased apoptosis. These research results provide rich data and theoretical basis for the diagnosis and treatment of diabetic sarcopenia.

## Introduction

Type 2 diabetes (T2DM) is a chronic metabolic disease characterized by high blood sugar, insulin resistance, and relative insulin deficiency (1). Skeletal muscle disorders are one of the common complications of diabetes, and they are closely related to acute and chronic hyperglycemia, insulin resistance, and hyperinsulinemia (2). Sarcopenia is a syndrome of muscle loss that occurs with aging and reduced physical activity. It is characterized by the reduction in the quantity, quality and strength of skeletal muscles. The occurrence of sarcopenia significantly increases the risks of falls, bedridden conditions and death in the elderly, seriously affecting the quality of life of patients and increasing the social and medical burdens. T2DM and sarcopenia often coexist in individuals. Studies have shown that among T2DM patients, 28% meet the diagnostic criteria for sarcopenia, and 58% exhibit varying degrees of sarcopenia manifestations (2). In recent years, single-factor studies on diabetic-induced sarcopenia have achieved remarkable results (3). However, due to the influence of various factors and complex biochemical processes, the pathogenesis of muscle lesions caused by T2DM remains unclear. Therefore, continuing to search for potential target genes and pathogenic pathways remains an effective strategy for exploring the pathogenesis of diabetic sarcopenia.

Proteomics conducts an overall analysis of the dynamic changes in the protein composition, expression, and post-translational modifications within the body. This enables a better understanding of the rules governing cellular life activities, as well as the changes in internal tissues and organs of the body, and the mechanisms of disease occurrence and development. This technology is increasingly being applied in the discovery of biomarkers, providing a powerful means for identifying proteins related to diseases (4). Over the past decade, numerous studies have provided information on the mechanisms underlying the continuous progression and deterioration of proliferative retinopathy (5), kidney diseases (6), and fatty liver (7) caused by diabetes through proteomics techniques. Previous research has found that ubiquitin is related to the expression patterns of muscle proteins in animal models (8). The important point is that some research teams have also conducted proteomics studies to identify the proteins involved in the pathogenesis of muscle atrophy-related traits, and to investigate the protein biomarkers with predictive and diagnostic value (9). Previous studies have confirmed that patients or animals with sarcopenia exhibit changes in muscle proteins (10–12). A recent study has also confirmed that lipid metabolism, particularly excessive fatty acid oxidation, may be a key factor in the progression of T2DM-related sarcopenia, and is also a common cause of the interrelationship between T2DM and sarcopenia (10). A study has found proteomic profiling of aged muscle tissues has shown shifts toward protein isoforms characteristic of a fast-to-slow transition process and an elevated number of oxidized proteins. In addition, a strong association between age and plasma values of growth differentiation factor 15 (GDF-15) has been described and serpin family A member 3 (serpin A3n) was more secreted by atrophied muscle cells (11). A study, based on proteomics methods, investigated the changes in skeletal muscle gene expression associated with age-related sarcopenia (12). These data suggest that altered muscle fibre type and metabolic rate along with increased cellular oxidative stress contribute to the age‐related decrease in muscle function. Due to the wide application of proteomics in exploring the pathogenic mechanism of sarcopenia, we used proteomics-specific statistical analysis to provide a comparative perspective, highlighting the subtle differences found in the skeletal muscles of diabetic rats, and providing new biological molecular landscapes and ideas for further exploration of the pathogenic mechanism of diabetic sarcopenia.

Autophagy and apoptosis are both important pathological and physiological mechanisms for regulating cell death. Some studies have explored the mechanisms of sarcopenia and the signaling pathways related to autophagy. These studies have pointed out that there is a connection between sarcopenia and mitochondrial autophagy, and have clarified the significant role of autophagy in the prevention and treatment of sarcopenia (13). However, the impact of autophagy-mediated protein degradation on the process of skeletal muscle reduction remains controversial (14). Autophagy regulates skeletal muscle cells differently under various circumstances, and both excessive and insufficient autophagy cause varying degrees of damage to skeletal muscle. This may be highly related to the functional state of autophagy or the relative proportion of autophagy and apoptosis in skeletal muscle during the aging process. Autophagy is closely related to apoptosis, and apoptosis is also an important mechanism that influences and regulates the occurrence and development of diabetes. Normal apoptosis is conducive to tissue renewal and the clearance of inflammatory cells (15). Previous studies have suggested that the apoptotic signal transduction is of great significance in protein degradation during muscle atrophy, and inhibiting the apoptosis of skeletal muscle cells has a protective effect on the muscles (15). In the presence of pro-apoptotic factors, skeletal muscle cells can produce anti-apoptotic factors to resist muscle loss, including high-intensity interval training and resistance training (16). Although there have been studies indicating that autophagy and apoptosis play important roles in the occurrence and development of sarcopenia, these studies are still controversial.

In our study, we were the first to apply proteomics to screen for differentially expressed proteins (DEPs) in the gastrocnemius muscle tissue of T2DM rats. We then performed GO and KEGG functional annotations and functional enrichment analyses on these DEPs, thereby further determining whether they might play a role in diabetic sarcopenia and the possible pathogenic pathways. Among the enriched pathogenic pathways, we have for the first time discussed the relationship between the occurrence of diabetic sarcopenia and autophagy and apoptosis of cells. This research may provide new ideas and theoretical basis for the diagnosis and treatment of diabetic sarcopenia.

## Materials and methods

### Experimental animals and grouping

Twelve healthy male Sprague-Dawley rats (provided by the Animal Experiment Center of Qingdao University, with production license number: SCXK (Zhejiang) 2019-0001), weighing 220-250g, were selected. All rats were randomly divided into the Control group (n=6) and the T2DM group (n=6). They were given free access to water and food, exposed to natural day-night light cycle, and maintained at a room temperature of 20-22°C. The control group was fed with a normal diet, while the T2DM group was given a high-sugar and high-fat diet (#MD12033; MediScience Diets Co., Ltd., Yangzhou, China) and was allowed to adapt to the diet for 4 weeks. All animal experimentation procedures were approved by the Experimental Animal Ethics Committee of Qingdao University (Approval No. 202301SD60202311100), and all animal experiments strictly followed the relevant ethical regulations.

### Modeling of T2DM rats

After 4 weeks of adaptive feeding, the rats in the T2DM group were fasted for 12 hours before drug injection. Streptozotocin (STZ, Aladdin Co., Ltd., Beijing, China) was freshly prepared and used immediately. Before use, it was dissolved in 0.1% citrate buffer (SSC, BIOISCO, 40 mg/kg) and placed in an ice bath. After weighing the rats, they were given a single intraperitoneal injection at a dose of 40mg/kg (17). After the modeling process, the control group of rats were fasted for 2 hours and given water. The rats in the experimental group were intravenously injected with the same volume of citrate buffer solution. After the injection, the Roche blood glucose monitor was used to measure the blood glucose levels of both groups 72 hours later. The criterion for successful modeling was: fasting blood glucose ≥ 16.7 mmol/L, with the result being consistent for three consecutive times (18).

### Sample collection

Six months after T2DM induction, Euthanasia was carried out by administering an intraperitoneal injection of pentobarbital euthanasia solution (Euthasol^®^) at a dosage of 100 mg/kg. The bilateral gastrocnemius muscles were completely removed through surgery. The muscles were washed with normal saline and the surface blood was absorbed. The gastrocnemius tissues were evenly divided into two parts. One part was placed in 4% paraformaldehyde for fixation for histological assessment, while the other part was frozen at -80°C for proteinomics, qRT-PCR and Western blot assays.

### Histological assessment

The gastrocnemius tissue was dehydrated, fixed, embedded in paraffin, sectioned, and stained with hematoxylin-eosin (HE) staining. After completion, the tissue was sealed with neutral gum and observed under a fully automatic scanner (3DHISTECH P250 FLASH, Beijing, China) to examine the pathological morphological changes of the gastrocnemius tissue and take photos. The steps of immunohistochemical staining are consistent with our previous study (17). In brief, take the gastrocnemius tissue for formaldehyde fixation, paraffin embedding, and consecutive sectioning. After dewaxing and hydration, the tissue is blocked, and the primary antibody is incubated at 4°C for 10 hours. The secondary antibody (goat anti-rabbit, 1:200, S0001, Affinity, USA) was incubated at 37°C for 1 hour. Used antibodies included Muscle Atrophy F-Box (MAFbx) antibody (1:100, DF7075, Affinity, USA) and ubiquitin-conjugating enzyme E2B (Ube2b) antibody (1:100, DF9999, Affinity, USA). Then, the reaction was performed with 2,5-diaminobenzidine for color development, followed by hematoxylin counterstaining and mounting with a cover slip. HampE and immunohistochemistry were observed using a Nikon E100 microscope (E100, Nikon, Japan), and scanning of images was performed using a panoramic scanner (3DHISTECH P250 FLASH, Beijing, China). The images were analyzed using the ImagePro Plus 8.0 software analysis system, and the positive expression level of the corresponding protein was determined based on the cumulative optical density (IOD) of the staining positive signal.

### Western blot

Take 50 mg of the gastrocnemius tissue stored at -80°C, extract the protein, and determine the protein concentration using the BCA method. Mix the concentration, prepare 12.5% gel, let it set at room temperature for 15 minutes, perform pre-electrophoresis at 80 V for 30 minutes, load the sample for electrophoresis at 120 V for 60 minutes, transfer the membrane at 300 mA for 80 minutes, and incubate with 5% skimmed milk for 2 hours. After washing with 1×TBST buffer (3 times, 10 minutes each), add the primary antibody at 4°C in the refrigerator overnight. The primary antibodies include Bax (1:10000, ab32503, Abcam, UK), Caspase-3 (1:2000, ab184787, Abcam, UK), p62 (1:4000, ab109012, Abcam, UK), and LC3-II/LC3-I (1:2000, 14600-1-AP, Proteintechgroup, USA). After washing with 1×TBST buffer (3 times, 10 minutes each time), add the secondary antibody (1∶5000) and incubate at room temperature on a shaker for 1 hour. Wash with TBST buffer 3 times, each time for 15 minutes. Add the ECL chemiluminescent agent (Thermo, USA) for 2 minutes and use the gel imaging system for imaging. Analyze the gray values of each band in the Western Blot images using Image J software.

### Real- time quantitative polymerase chain reaction

The experimental procedures were consistent with those in previous literature (17). In brief, 50 mg of gastrocnemius tissue was taken, and total RNA in the tissue was extracted using Trizol reagent. The total mRNA was used as a template for reverse transcription to cDNA, which was then placed in a fluorescence quantitative PCR instrument for amplification. The reaction conditions were set according to the instructions. The Ct values of each group of genes were obtained, and the 2^-ΔΔCt^ method was used for analysis and calculation. GAPDH was used as the internal control. Finally, the relative expression level of the target gene was obtained. The primer sequences are shown in **Table 1**.

**Table 1:** Primer information of qRT-PCR gene.

Gene	Sequence (5’-3’)
Bax	Bax-F: ATCATGAAGACAGGGGCCTTTTT
	Bax-R: TGCTCGATCCTGGATGAAACTAG
Caspase-3	Caspase-3-F: CTTGTAACATGCAACTAAGAGCTC
	Caspase-3-R: CAGGGCCATGAATGTCTCTCTG
p62	p62-F: GGTGTCTGTGAGAGGACGAGGAG
	p62-R: TCTGGTGATGGAGCCTCTTACTGG
LC3	LC3-F: CCAGGACAAGCAGGCAGATGAAG
	LC3-R: CAGGCTTTCGTCTCTTCCACCATC
GAPDH	Gapdh-F: CTGCCTTCTCTTGTGACAAAGTG
	Gapdh-R: TTGATGACCAGCTTCCCATTCTC

qRT-PCR: real-time quantitative polymerase chain reaction; Bax: Bcl-2 associated X protein; Caspase-3: Cysteine-dependent aspartate-specific protease-3; p62: sequestosome-1; LC3: microtubule-associated proteins light chain 3.

### Proteomics analysis

All samples were delivered to LC-BIO Technologies Co., Ltd. (Hangzhou, Zhejiang, China) for proteomics analysis. Briefly, the proteins were extracted by tissue homogenization using a tissue lyser(60 Hz, 2 min), followed by 15-min centrifugation (20,000 × g, 4°C). Quality control of protein extraction was conducted using the Bradford method and SDS-PAGE. Then, the obtained proteins were digested with trypsin at the 50:1 ratio of protein to trypsin. After digestion, the peptides were labeled with Tandem Mass Tag (TMT; ThermoFisher Scientific, USA). Equal quantities of peptides from each sample were mixed and fractionated using a 3.5 μm 4.6 × 150 mm Agilent ZORBAX 300Extend-C18 column on the UltiMate™ 3000 Binary Rapid Separation System (Thermo Fisher Scientific, USA). The gradient elution was performed using monitoring elution peaks at 214 nm, and fractions were collected every minute (19). The obtained fractions were freeze-dried. Afterward, the dried peptide samples were redissolved and separated using the EASY-nLC™ 1200 system (Thermo Fisher Scientific, USA). The separated peptides were ionized using a nano-ESI and then transferred to Orbitrap Exploris™ 480 mass spectrometer (MS; Thermo Fisher Scientific, USA) for DDA mode detection. MaxQuant (version 2.1.4.0) software was used to analyze the TMT-plexed MS/MS raw data. Statistical analysis was performed using R software (version 4.0.0). Proteins were considered statistically different when the p value was <0.05 and the fold-change was >1.2. OmicStudio tools at https://www.omicstudio.cn/tool were used to draw principal component analysis chart, volcano plot and biological process enrichment scatter plot (20).

### Statistical analysis

All data analyses were performed using SPSS 21.0 (IBM, Armonk, NY) and GraphPad Prism 8.0 (La Jolla, CA). All data were tested for normal distribution using the Quantile-Quantile Plot. Measurement data in accordance with normal distribution were described by means ± standard deviation, and comparison between the two groups was conducted by Student’s t-test. A P value < 0.05 indicated statistical significance.

## Results

### Histological assessment

**Figure 1:**
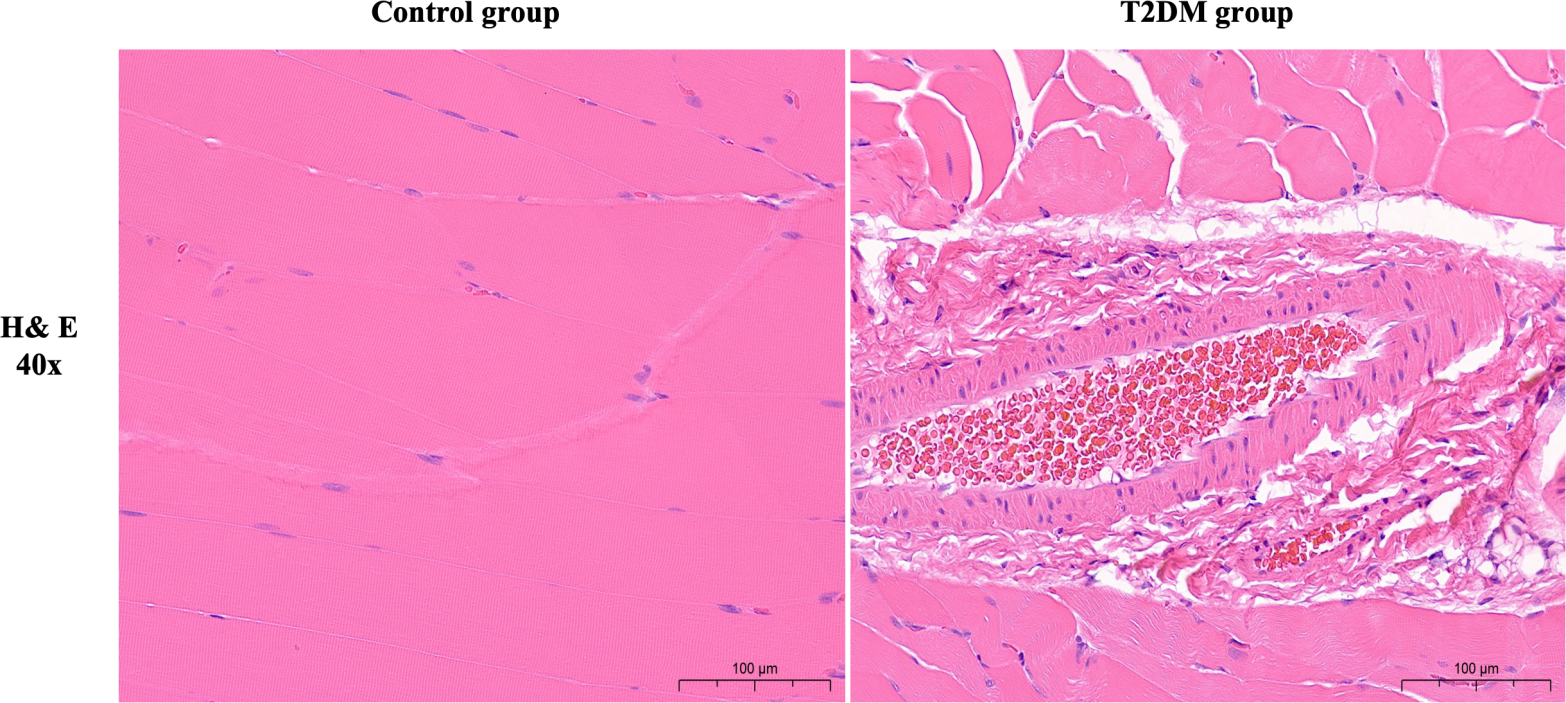
Representative images of Hematoxylin and eosin (H&E) staining (40X). Scale bars depict 100µm.

**Figure 2:**
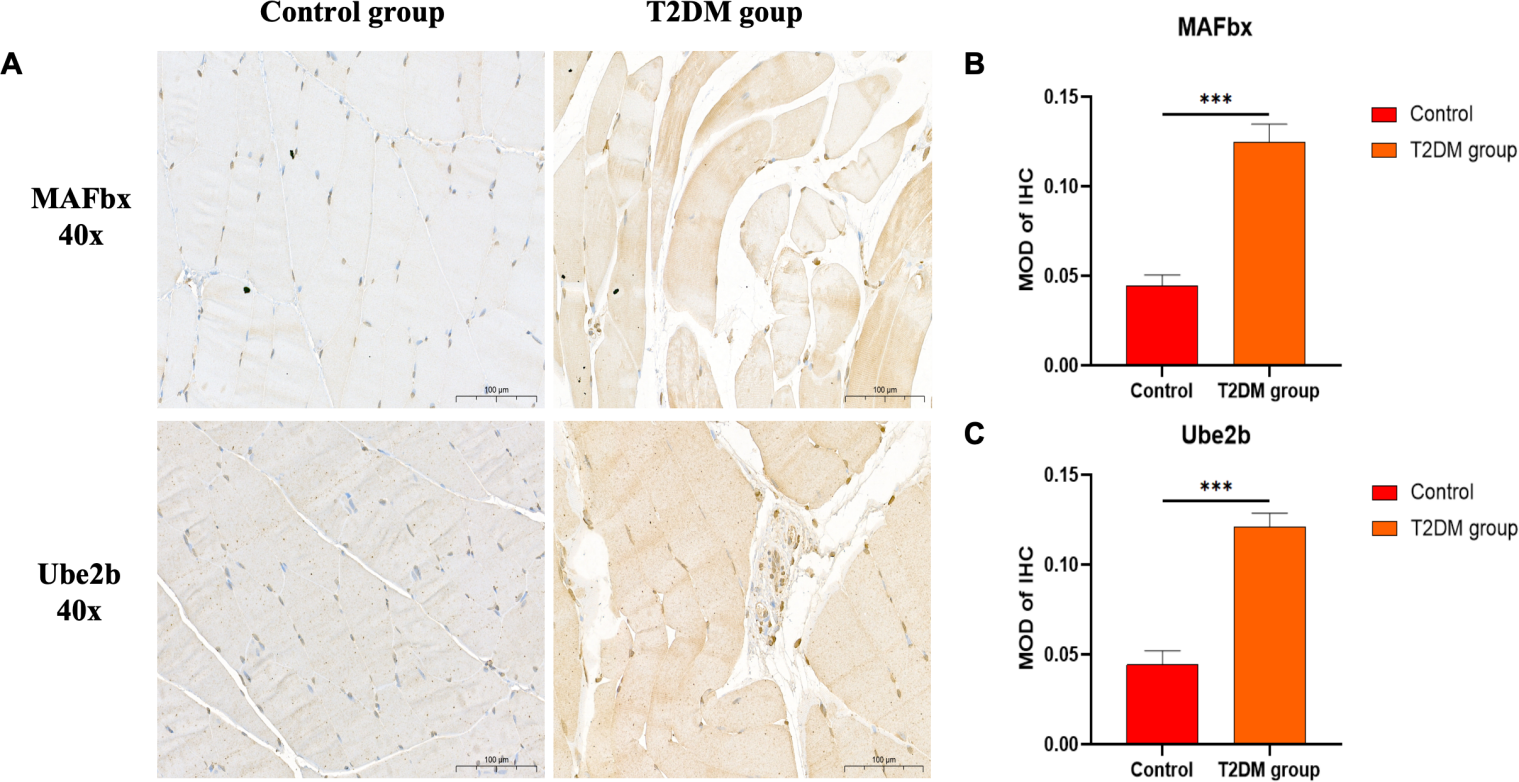
Representative images of immunohistochemical staining. **(A)** MAFbx and Ube2b (40X). Comparison of semi-quantitative MOD after immunohistochemical staining. MOD is the ratio of cumulative optical density value (IOD) and positive area (AREA). **(B)** MAFbx (40X). **(C)** Ube2b (40X). ***p < 0.001 versus Control group. The values are presented as means, with the error bars depicting the standard deviation. Scale bars depict 100µm.

Rats in the T2DM group with random blood glucose > 16.7 mmol/L after modeling showed significant symptoms of polydipsia and polyphagia. They also gained weight slowly compared with the control group. The detailed results are shown in **Additional File 5**. The results of HE staining showed that the muscle cells in the normal group were plump, with small cell spacing and no abnormal morphology; in the T2DM group, there was significant atrophy, a reduction in cross-sectional area, increased cell spacing and sparseness, as well as signs of hemorrhage and inflammatory infiltration (**Figure 1**). The immunohistochemical staining results showed that, compared with the control group, the expression levels of MAFbx and Ube2b proteins in the gastrocnemius muscle of T2DM rats significantly increased (P < 0.001), indicating that the gastrocnemius muscle of T2DM rats was significantly atrophied (**Figure 2**). These results all indicate that significant pathological changes occurred in the gastrocnemius tissue of T2DM rats.

### Proteomics analysis

**Figure 3:**
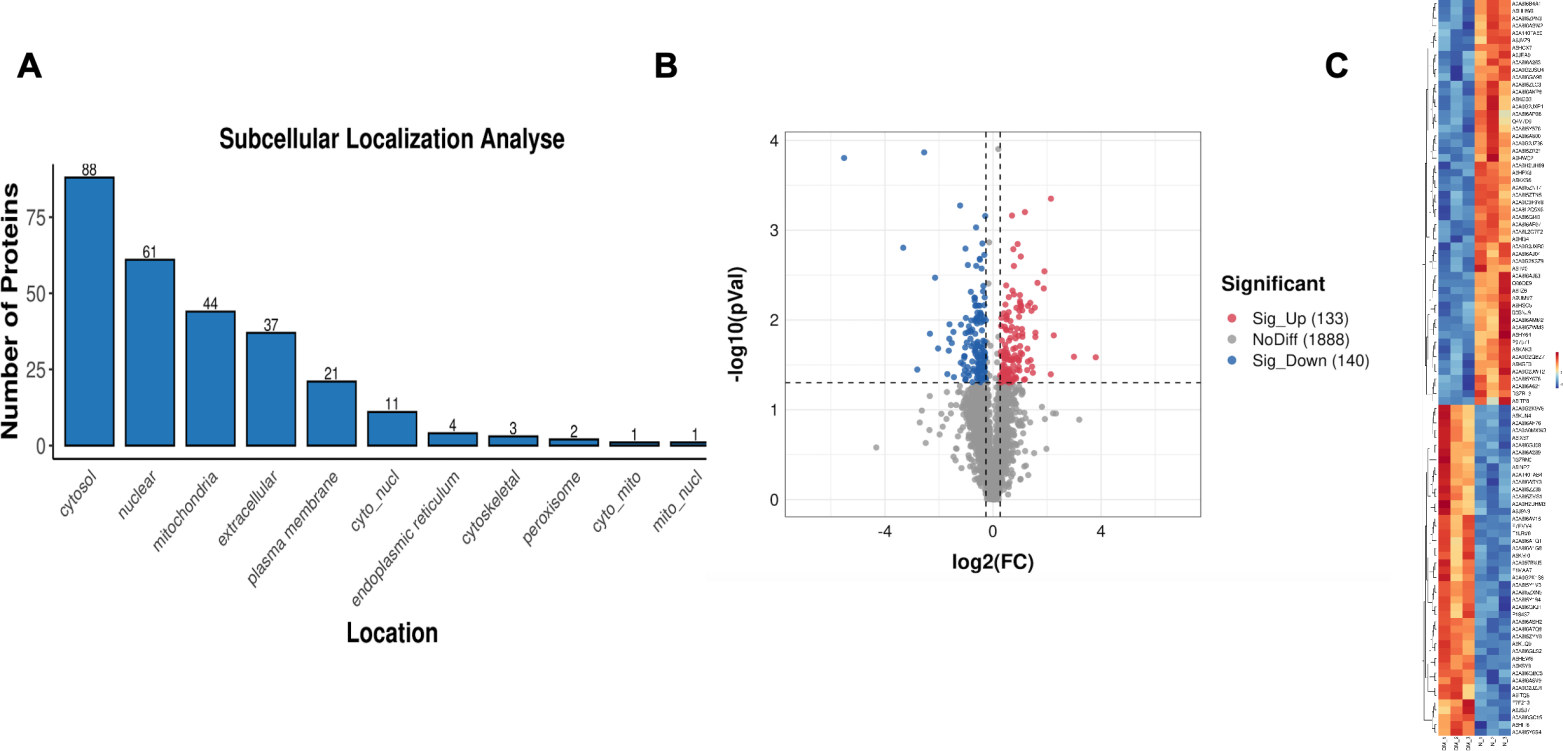
**(A)** Subcellular localization map of differentially expressed proteins. **(B)** Differentially expressed protein quantitative volcano plot. **(C)** Heatmap of differentially expressed proteins.

Proteomics analysis in the gastrocnemius muscle tissue of T2DM group rats identified 273 differentially expressed proteins (DEPs), among which 133 were upregulated and 140 were downregulated (**Supplementary Table 1**). The representative DEPs that showed significant changes (Top 10) are shown in **Table 2**. Subcellular structure analysis revealed that most of the DEPs were expressed in extracellular (35 DEPs), cytosol (34 DEPs), nucleus (34 DEPs), plasma membrane (22 DEPs), and mitochondria (15 DEPs) (**Figure 3A**). The red dots in the volcano plot represent up-regulated proteins, the blue dots represent down-regulated proteins, and the black dots represent proteins with no significant difference (**Figure 3B**). The hierarchical clustering analysis heatmap visually presents the global expression changes of multiple samples and multiple proteins, and shows the clustering relationships of the expression levels of multiple samples or multiple proteins (**Figure 3C**).

**Table 2:** Two comparison groups up-regulated and down-regulated DEPs (Top10).

Grouping situation	Up-regulated (Top10)		Down-regulated (Top10)	
	DEPs	FC	DEPs	FC
T2DM vs NG	rCG_19945	1.82	Uqcr11	0.17
	LOC100912677	1.28	ND2	0.02
	Cacnb2	1.22	Fhod1	0.43
	Col6a6	1.40	Hspb2	0.82
	Spcs2	1.64	Mapt	0.65
	Kng2	1.32	Cops2 rCG_26697	0.76
	Lamc1	1.26	Tnnt3 rCG_47210	0.10
	rCG_33140	1.87	G3bp1	0.49
	Fabp4	1.62	Prdx1l1	0.80
	Hspg2	1.31	Map2k2	0.71

DEPs: Differentially expressed proteins; FC: fold change.

### Bioinformatics analysis of differentially expressed proteins

**Figure 4:**
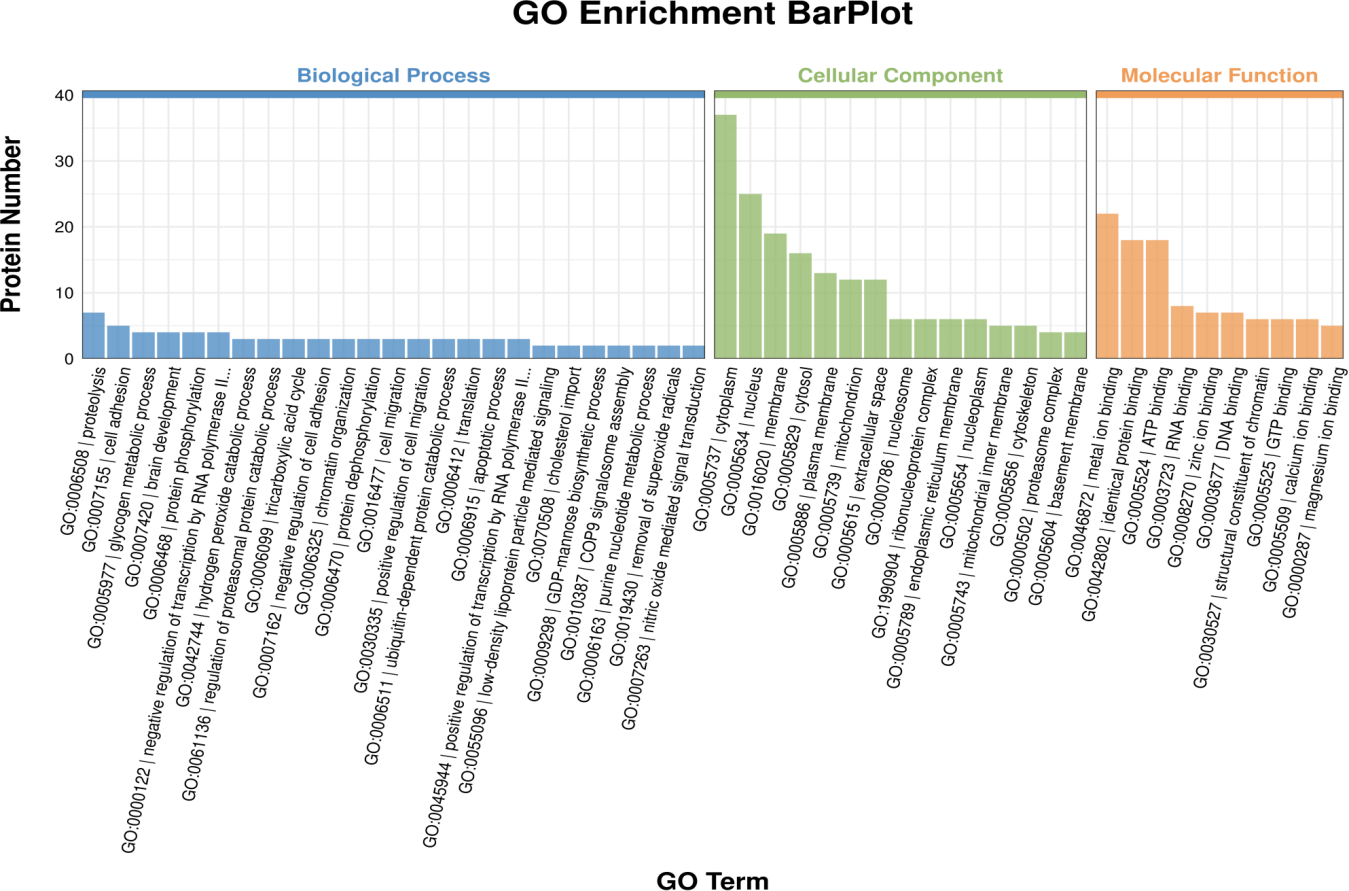
Gene ontology (GO) secondary functional classification.

**Figure 5:**
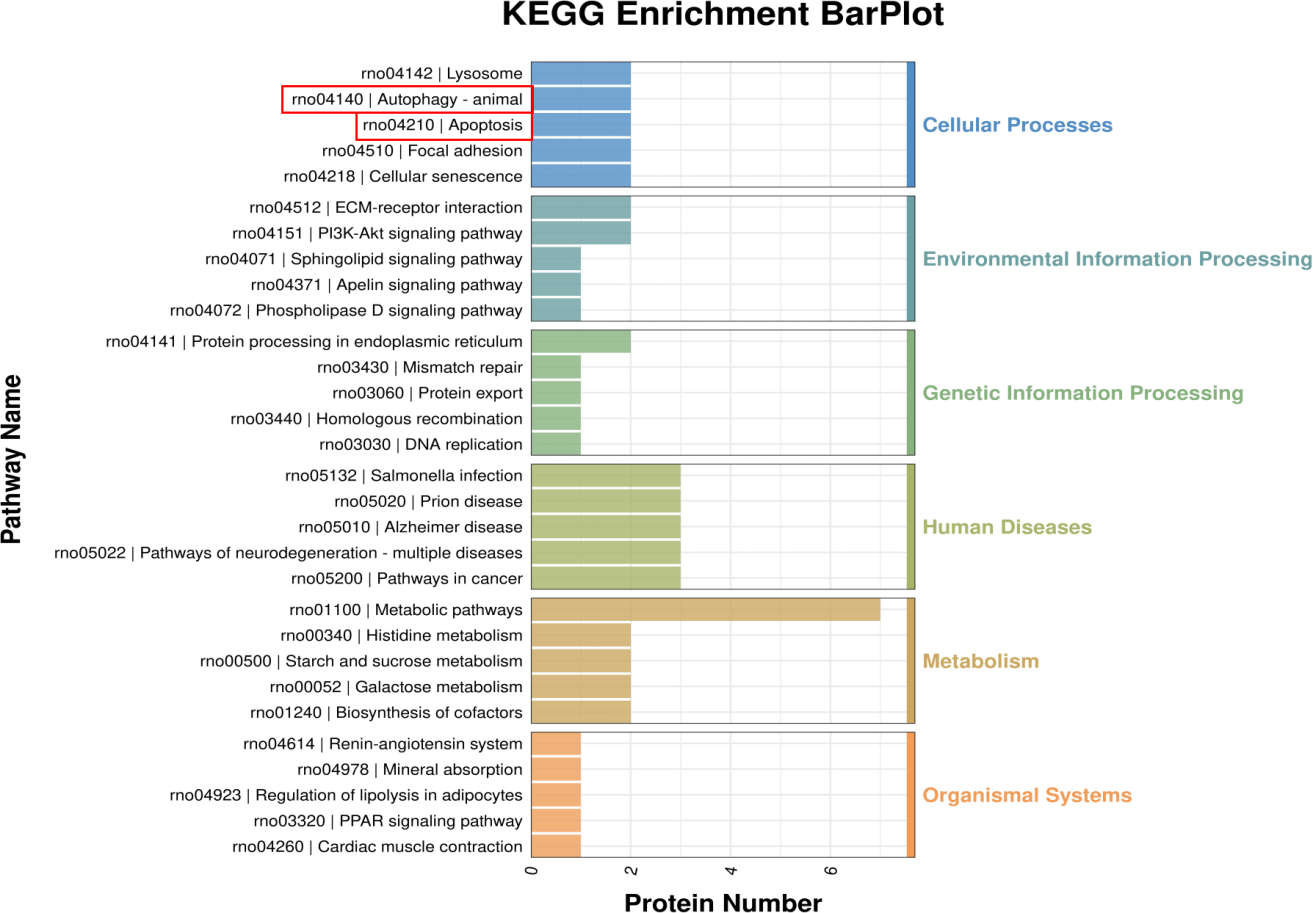
KEGG enrichment analysis of differentially expressed proteins.

Compared with the control group, in the GO enrichment analysis of differentially expressed proteins in the T2DM group, the main biological processes involved included glycogen metabolic process, hydrogen peroxide catabolic process, low-density lipoprotein particle mediated signaling and cholesterol import, etc.; the main cellular components involved included nucleosome, proteasome regulatory particle, lid subcomplex and proteasome accessory complex, etc.; the main molecular functions involved included structural constituent of chromatin, oxidoreductase activity, acting on the CH-OH group of donors, NAD or NADP as acceptor, glycosphingolipid binding and thioredoxin peroxidase activity, etc. (**Supplementary Table 2**, **Figure 4**). The KEGG enrichment analysis (**Supplementary Table 3**) revealed that the signaling pathways involved in DEPs were mainly Histidine metabolism, Starch and sucrose metabolism, and Galactose metabolism (p < 0.01) (**Figure 5**, **Table 3**). Among them, the pathway with the highest number of DEPs is metal ion binding, with 22 DEPs expressed. Additionally, the main Cellular Processes that DEPs are involved in include autophagy and apoptosis signaling pathways. These two pathways may be closely related to the occurrence of diabetic sarcopenia.

**Table 3:** Pathways of major enrichment of 153 DEPs (p < 0.05).

Pathway ID	Pathway description	Matching IDs	Gene name	P-value
rno00340	Histidine metabolism	A0A0G3F9V8, A0A0G2JSI1	Carnmt1,Aldh9a1	0.002469958
rno00500	Starch and sucrose metabolism	A0A9K3Y8H8, Q6P7A9	Ugp2,Gaa	0.002820029
rno00052	Galactose metabolism	A0A9K3Y8H8, Q6P7A9	Ugp2,Gaa	0.003790839
rno04512	ECM-receptor interaction	F1MAA7, A0A8I5ZQ25	Lamc1,Lamb1	0.014537679
rno05222	Small cell lung cancer	F1MAA7, A0A8I5ZQ25	Lamc1,Lamb1	0.028468665
rno05146	Amoebiasis	F1MAA7, A0A8I5ZQ25	Lamc1,Lamb1	0.031603131
rno05132	Salmonella infection	A0A0G2JZ38, A0A8I5ZLZ9, D3Z8L7	Fhod1,Nckap1,Rras	0.0368518
rno05145	Toxoplasmosis	F1MAA7, A0A8I5ZQ25	Lamc1,Lamb1	0.049155574

### The expression of autophagy proteins and genes in the gastrocnemius muscle tissue of T2DM rats

**Figure 6:**
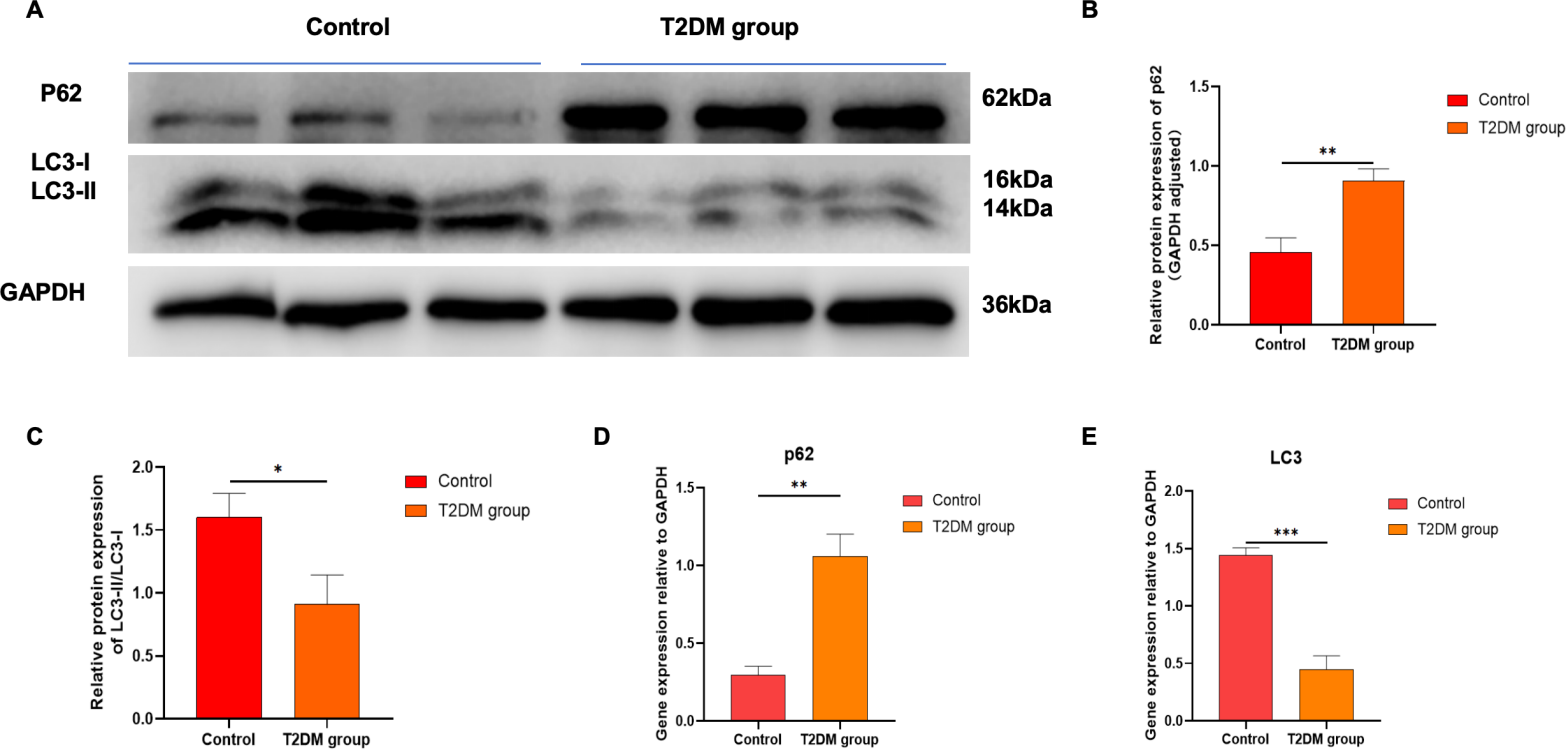
**(A–C)** The protein levels of P62 and LC3II/I were detected by Western blot. The full and uncropped Western blot images can be found in **Additional File 4**. **(D)** The mRNA expression level of P62. **(E)** The mRNA expression level of LC3. *p < 0.05 versus Control group. **p < 0.01 versus Control group. ***p < 0.001 versus Control group. The values are presented as means, with the error bars depicting the standard deviation.

The expression of key proteins in the autophagy pathway was detected by Western blotting (**Figures 6A–C**). Compared with the control group, the expression of p62 protein in the gastrocnemius tissue of T2DM rats significantly increased (P < 0.01), and the ratio of LC3II/I protein decreased (P < 0.05). Compared with the control group, the mRNA level of LC3 in the gastrocnemius tissue of T2DM rats significantly decreased (P < 0.001), and the mRNA level of p62 significantly increased (P < 0.01), and the differences were statistically significant (**Figures 6D, E**). These results indicate that the autophagy level in the gastrocnemius tissue of T2DM rats is significantly decreased.

### The expression of apoptotic proteins and genes in the gastrocnemius muscle tissue of T2DM rats

**Figure 7:**
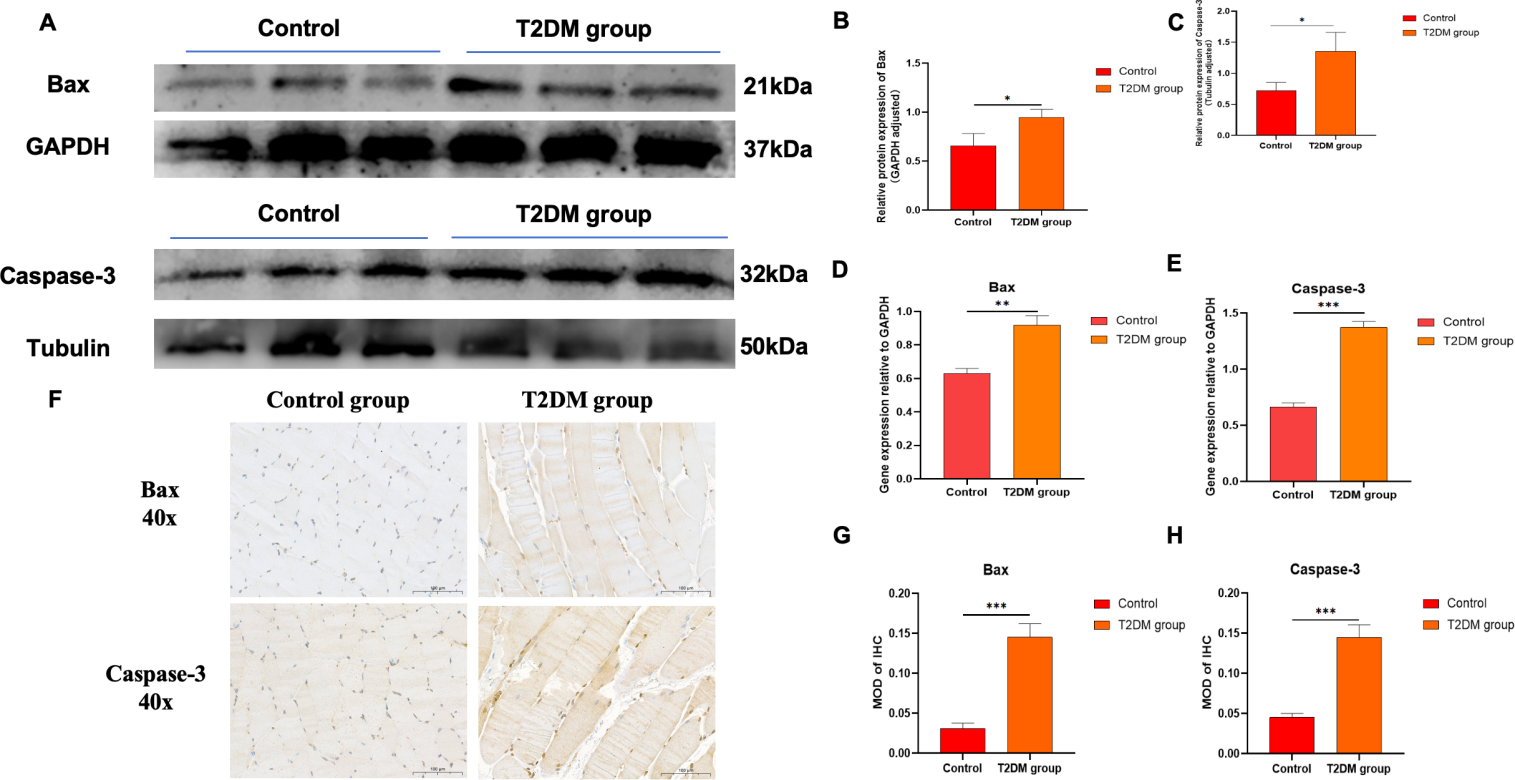
**(A–C)** The protein levels of Bax and Caspase-3 were detected by Western blot. The full and uncropped Western blot images can be found in **Additional File 4**. **(D)** The mRNA expression level of Bax. **(E)** The mRNA expression level of Caspase-3. **(F)** Typical immunohistochemical staining images of Bax and Caspase-3. **(G)** MOD value of Bax in immunohistochemical staining. **(H)** MOD value of Caspase-3 in immunohistochemical staining. *p < 0.05 versus Control group. **p < 0.01 versus Control group. ***p < 0.001 versus Control group. The values are presented as means, with the error bars depicting the standard deviation.

The results of Western blotting assay (**Figures 7A–C**) showed that compared with the control group, the protein expression levels of Bax and Caspase-3 in the gastrocnemius muscle tissue of T2DM rats were significantly increased, and the difference was statistically significant (p < 0.05); compared with the control group, the mRNA levels of Bax and Caspase-3 in the gastrocnemius muscle tissue of T2DM rats were significantly increased, and the difference was statistically significant (P < 0.001) (**Figures 7D, E**). The results of immunohistochemical staining (**Figures 7F–H**) showed that, compared with the control group, a large amount of brown substances were observed in the cytoplasm of the gastrocnemius muscle tissue of the T2DM group rats. The positive expressions of Bax and Caspase-3 increased, and the difference was statistically significant (P < 0.001).

## Discussion

Sarcopenia is a progressive and systemic loss of skeletal muscle mass and function with age. Endocrine factors such as diabetes are one of the reasons for the occurrence and development of sarcopenia (21). Studies have shown that the prevalence of sarcopenia in patients with T2DM is as high as 18% (22), and the incidence is 1.5 times that of non-diabetic patients (23). The etiology and pathogenesis of sarcopenia caused by T2DM are very complex, including increased insulin resistance, impaired function of muscle satellite cells, inflammatory responses in skeletal muscles, and damage to microvessels in skeletal muscles, etc. (24, 25). Insulin resistance in skeletal muscle is a major factor in the onset of T2DM. Skeletal muscle is one of the most important target organs for insulin action. Insulin resistance can lead to muscle atrophy and loss of function (5). Insulin-stimulated glucose uptake into skeletal muscle has a significant impact on the body’s glucose homeostasis and can exacerbate muscle atrophy (25). More and more studies have explored the mechanism by which T2DM causes sarcopenia, but as of now, the results of various studies still have some disputes and differences. Over the past decade, numerous studies have utilized proteomics techniques to provide insights into the mechanisms underlying the continuous progression and deterioration of proliferative retinopathy (5), kidney disease (6), and fatty liver (7) caused by diabetes. However, the correlation between protein profiles and the severity of diabetic sarcopenia has not been investigated.

The proteome refers to all the corresponding proteins expressed by the genes of a cell or tissue at a specific time and space, including various subtypes and protein modifications (26). Proteomics analyzes the dynamic changes of protein components within a cell from an overall perspective, understands the interactions and connections between proteins, and thereby reveals the functions of proteins and the laws of life activities (27). Proteins may be disturbed by pathological conditions, especially when the disease “is approaching”. Therefore, mapping the protein profiles of the gastrocnemius muscle and understanding their changes and patterns during the muscle lesion process induced by T2DM is one of the main goals of this study. Based on proteomics analysis, we found that a total of 273 DEPs were identified in the gastrocnemius muscle tissue of T2DM rats. These DEPs may be potential biomarkers for muscle lesions caused by T2DM. These abnormalities of DEPs will have an impact on signal transduction through various pathways. The results of KEGG enrichment analysis include 94 potential pathogenic pathways. In this study, we first analyzed and verified the roles of autophagy and apoptosis pathways in diabetic sarcopenia based on proteomics results, and conducted a preliminary exploration.

Over the years, numerous studies have explored the pathogenic mechanisms of age-related muscle mass reduction through proteomics. Among various hypotheses, the protein homeostasis involving a series of processes such as protein synthesis, folding, transport, and degradation has been regarded as the main mechanism leading to age-related muscle mass loss (28). At this point, when the reduction in protein synthesis and/or the increase in protein degradation reach the extent that disrupts the protein balance and homeostasis, muscle atrophy occurs, and the oxidative capacity of skeletal muscle in the elderly increases while the glycolytic capacity decreases (29). We also attempted to explore through multi-omics approaches such as transcriptomics and metabolomics, but the changes in gene expression do not always reflect the changes in protein levels or activities. Proteomics based on mass spectrometry has successfully been applied to study the crude extracts and subcellular fractions of muscle tissues from aging animals and humans, in order to identify novel aging-related marker proteins. Proteomics has revealed important molecular events in mitochondria during the onset of age-related sarcopenia. The consumption of mitochondrial energy metabolism proteins and neuromuscular junction proteins is most significantly correlated with sarcopenia, suggesting that a treatment that simultaneously stimulates mitochondrial generation and inflammation may have the potential to treat sarcopenia (30). Furthermore, in terms of the mechanism by which high-intensity interval training improves muscle strength in the elderly, the proteomic results compared with moderate-intensity continuous training revealed that mitochondrial function, apoptosis, regeneration, and antioxidant factors were involved in the process of improving skeletal muscle strength (31).

Proteomics techniques have also been used to identify biomarkers for sarcopenia. To date, hundreds of protein biomarkers with high risk and potential diagnostic applications have been identified from cross-sectional studies (32). Based on this, several proteins have been used to predict the occurrence or severity of sarcopenia. A longitudinal study found that peripheral tumor necrosis factor-α might be a marker for the progression of sarcopenia in patients with hip fractures (33). Some age-related proteins have been identified at present, which are related to muscle metabolism, ion handling and the response of cells to stress (34). A label-free quantitative proteomics study was conducted to investigate the mechanism of human skeletal muscle aging. This study found that 35 DEPs were associated with muscle aging during the aging process, and some proteins involved in energy metabolism and contraction proteins were related to muscle aging. It may regulate the connectivity between neurons and muscle fibers by influencing the synthesis and degradation of proteins in the muscles, which is crucial for understanding the physiological impact of protein homeostasis disruption caused by aging (35). Therefore, in our study, identifying these 273 DEPs may provide a new understanding of the origin of structural and functional changes in skeletal muscle caused by high blood sugar. The subsequent experimental verification of key DEPs is also one of the focuses of our research.

Our research has revealed that the autophagy level in the gastrocnemius muscle tissue of T2DM rats has significantly decreased, which may be related to the occurrence of diabetic sarcopenia. Previous studies have made certain contributions to the relationship between sarcopenia and autophagy. Some studies have explored the mechanisms of sarcopenia and the signaling pathways related to mitochondrial autophagy. These studies have pointed out that there is a connection between sarcopenia and mitochondrial autophagy, and have clarified the important role of mitochondrial autophagy in the prevention and treatment of sarcopenia (36). Autophagy is an evolutionarily conserved process by which cellular components are self-degraded through autophagosomes, and the degradation products are delivered to lysosomes, thereby preventing the accumulation of cellular metabolic waste (37). However, the impact of autophagy-mediated protein degradation on the process of skeletal muscle reduction remains controversial, which may be highly related to the functional state of autophagy or the relative proportion of autophagy and apoptosis in skeletal muscle during aging. As a protective mechanism against different forms of stress, the basal activation of autophagy is necessary for cellular homeostasis, and the moderate activation of autophagy, mediated through multiple signaling pathways, may have a significant inhibitory effect on skeletal muscle reduction. Autophagy regulation of skeletal muscle cells has different effects under different circumstances. However, both excessive and insufficient autophagy cause varying degrees of damage to skeletal muscle. Autophagy has a dual nature. Appropriate autophagy has a protective effect on muscle tissue during normal aging process, while excessive activation of autophagy causes damage to the muscles (38). In the model of sarcopenia caused by aging animals, the changes in autophagy related to aging still remain controversial. Activation of autophagy in the skeletal muscles of aged mice, and similar results were reported in dogs (10). However, another study reached the opposite conclusion, that compared with adult rats, the expression of autophagy-specific protein LC3 in the skeletal muscles of elderly rats was decreased (11). Autophagy actually maintains muscle mass and promoting basal autophagy can protect against age-related muscle dysfunction. On the contrary, autophagy inhibition leads to the loss of muscle strength and causes atrophy of muscle fibers in the elderly (12). Current evidence suggests that excessive activation of autophagy can also lead to atrophy of skeletal muscle, which may be a factor contributing to age-related loss of skeletal muscle. Whether autophagy is protective or harmful seems to be determined by the level and duration of the autophagy process. Although the appropriate induction and normal regulation of the autophagy process are necessary to maintain skeletal muscle mass, the basic molecular mechanisms remain largely unknown. Therefore, how to properly control the intensity of autophagy and when to do so are of great significance in the treatment of sarcopenia.

Apoptosis is a process by which cells end their lives, involving many genes and having a very complex regulatory mechanism. Previous studies have suggested that the apoptotic signal transduction plays a significant role in protein degradation during muscle atrophy, and inhibiting apoptosis of skeletal muscle cells has a protective effect on muscles (13). In the presence of pro-apoptotic factors, skeletal muscle cells can produce anti-apoptotic factors to resist muscle loss, including high-intensity interval training and resistance training (14). Similarly, we verified the conclusion that cell apoptosis significantly increased in the gastrocnemius muscle tissue of T2DM rats through proteomics data. Studies have shown that the apoptosis of rat muscle cells can be reduced through the following methods: lifelong calorie restriction can promote the expression of IL-15, where IL-15 is a cytokine in the muscle anabolic pathway and can slow down the muscle loss and cell apoptosis in aged rats. This may be partially achieved by inhibiting the apoptosis triggered by TNF-α (39). The TNF-α signal transduction mainly activates the ubiquitin-proteasome pathway, thereby enhancing the protein breakdown ability in skeletal muscle cells (40). After training, the mass of the gastrocnemius muscle and body weight of rats, as well as the expressions of proteins such as Bcl-2 and SIRTl, significantly increased. Exercise training can inhibit age-induced apoptosis of skeletal muscle cells, thereby preventing muscle loss in aged rats (41). Studies have confirmed that melatonin has the ability to act as an anti-apoptotic molecule in skeletal muscle cells, which may provide a possible therapeutic strategy for muscle disorders involving dysregulated apoptosis (42). Therefore, based on our research results, the application of hypoglycemic drugs and other medications may have the effect of preventing or treating diabetic sarcopenia, although these evidences are currently not sufficient. This is also a new area that we need to study in the future.

Based on proteomics, this study conducted a preliminary investigation on the proteins in the gastrocnemius muscles of T2DM rats, obtaining some meaningful experimental results. However, there are still many issues to be resolved. Firstly, this experiment only preliminarily explored the possible pathways of diabetic sarcopenia at the protein level. The key DEPs and their possible molecular mechanisms require further experimental exploration. Among the 273 DEPs, we further screened out the key proteins that might have the potential to serve as markers for diagnosing diabetic sarcopenia. However, the identification of the target proteins and the validation of the subsequent related signaling pathways are currently lacking. We expect the results of this study to provide clues and ideas for other researchers in this field. Secondly, the sample size of this study is relatively small. Different studies use different models and sample sizes, which may lead to inconsistent conclusions. Therefore, more research is needed to verify these findings. In the future, it is still necessary to further expand the sample size for verification. Thirdly, proteomics based on animal samples still cannot fully simulate the pathological and physiological states of human diseases. We expect that subsequent experiments will be able to be verified in human samples. Fourthly, our research findings indicate that sarcopenia caused by T2DM may be related to autophagy and apoptosis, but the specific regulatory mechanisms and the involved pathways remain unclear, especially the interactions between DEPs and signaling pathways. Finally, TMT proteomics cannot provide absolute quantitative information. During the experiment, there may be labeling deviations, leading to errors in quantitative results. Therefore, the combined analysis of multiple omics is crucial in the future.

## Conclusions

This study utilized proteomics technology to screen out 273 DEPs in the gastrocnemius muscle of T2DM rats. Bioinformatics analysis and exploration of potential pathogenic pathways were conducted on these proteins. By verifying proteins related to the pathways of autophagy and apoptosis, we were the first to confirm through proteomics that sarcopenia caused by T2DM may be associated with decreased autophagy and increased apoptosis. The results of this study provide rich data and theoretical basis for the diagnosis and treatment of diabetic sarcopenia.

## Data Availability

The datasets presented in this study can be found in online repositories. The names of the repository/repositories and accession number(s) can be found in the article/**Supplementary Material**.
