# Supplementary material for: Proteomic analysis confirmed that the occurrence of diabetic sarcopenia is related to autophagy and apoptosis
Source: Front Endocrinol (Lausanne). 2026 Jan 5;16:1656035. doi: 10.3389/fendo.2025.1656035 (PMC12812756; doi:10.3389/fendo.2025.1656035)
Supplement: Supplementary file 4 [file Table4.pdf]

Raw data of Western blot: Full, uncropped blot images

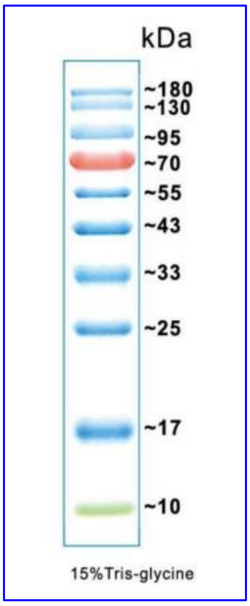

Share-bio#WB009
Protein Standards used in the study.

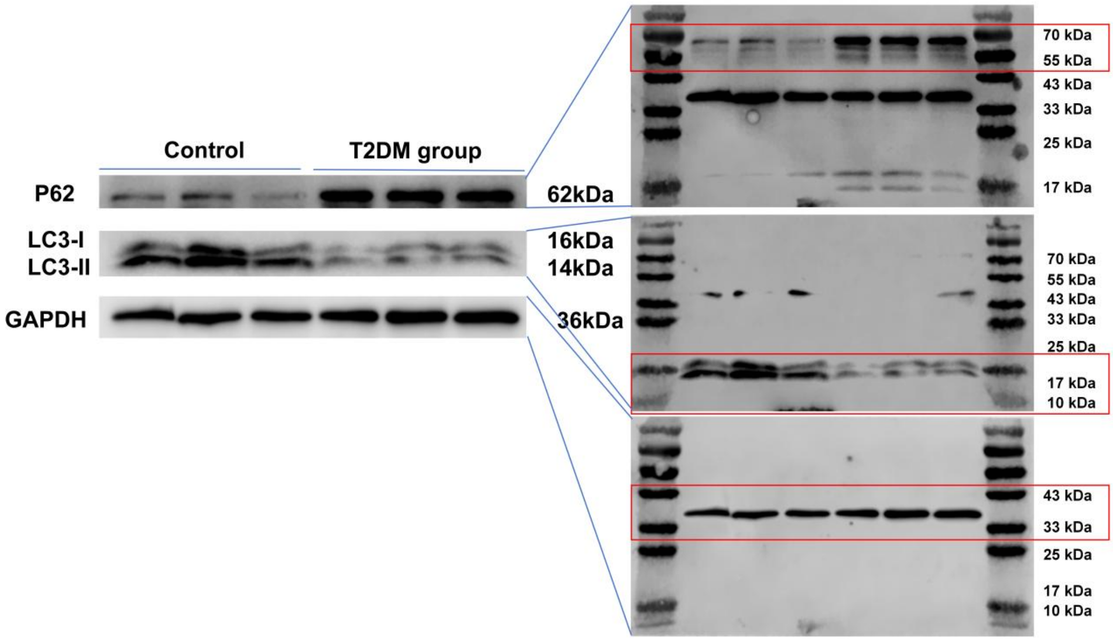

Fig. S1 Full-length blots for Fig. 6A.

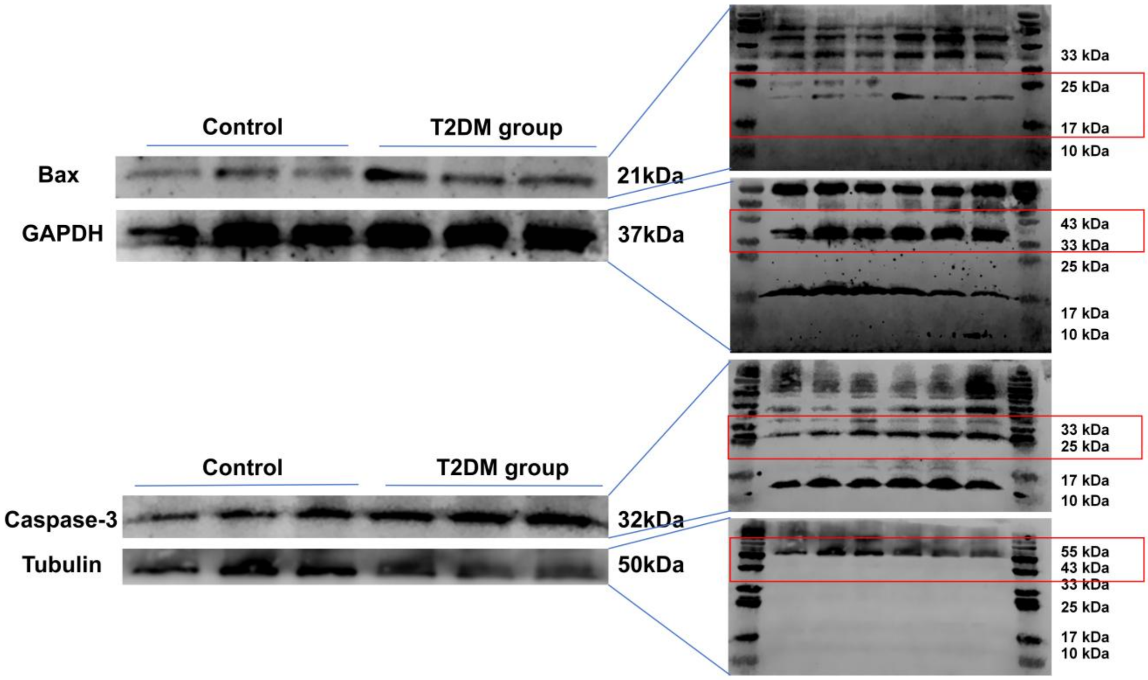

Fig. S2 Full-length blots for Fig. 7A.
