# Supplementary material for: Proteomic analysis confirmed that the occurrence of diabetic sarcopenia is related to autophagy and apoptosis
Source: Front Endocrinol (Lausanne). 2026 Jan 5;16:1656035. doi: 10.3389/fendo.2025.1656035 (PMC12812756; doi:10.3389/fendo.2025.1656035)
Supplement: Supplementary file 5 [file Table5.pdf]

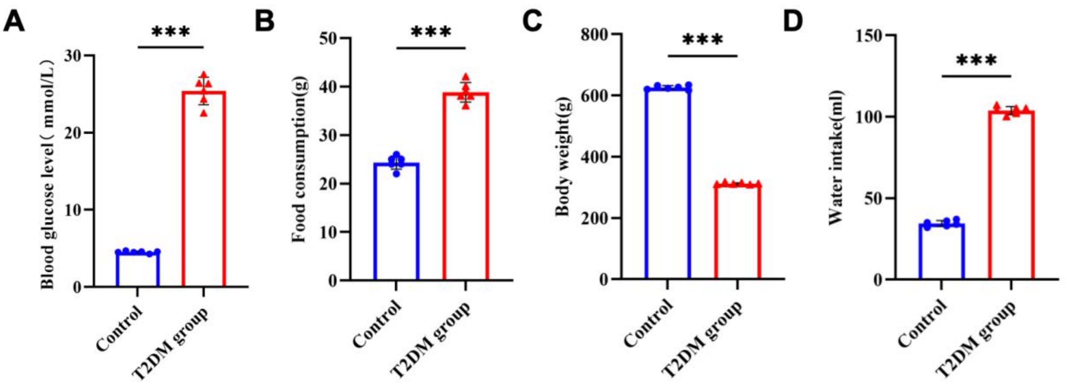

Additional file 5. Evaluation of a rat model type 2 diabetes. (A) Blood glucose level. (B) Food consumption. (C) Body weight. (D). Water intake;
